# Minimal ablative margin (MAM) assessment with image fusion: an independent predictor for local tumor progression in hepatocellular carcinoma after stereotactic radiofrequency ablation

**DOI:** 10.1007/s00330-019-06609-7

**Published:** 2020-01-30

**Authors:** Gregor Laimer, Peter Schullian, Nikolai Jaschke, Daniel Putzer, Gernot Eberle, Amilcar Alzaga, Bruno Odisio, Reto Bale

**Affiliations:** 1 Department of Radiology, Interventional Oncology-Microinvasive Therapy (SIP), Medical University Innsbruck, Anichstr. 35, 6020 Innsbruck, Austria; 2 Department of Internal Medicine I, Gastroenterology, Hepatology, Endocrinology and Metabolism, Medical University Innsbruck, Anichstr. 35, 6020 Innsbruck, Austria; 3 Siemens Healthineers, Siemensstraße 3, 91301 Forchheim, Germany; 4 Division of Diagnostic Imaging, Department of Interventional Radiology, The University of Texas MD Anderson Cancer Center, Houston, TX USA

**Keywords:** Ablation techniques, Radiofrequency ablation, Carcinoma, hepatocellular, Tomography, X-ray computed, Treatment outcome

## Abstract

**Objectives:**

To assess the minimal ablative margin (MAM) by image fusion of intraprocedural pre- and post-ablation contrast-enhanced CT images and to evaluate if it can predict local tumor progression (LTP) independently. Furthermore, to determine a MAM with which a stereotactic radiofrequency ablation (SRFA) can be determined successful and therefore used as an intraprocedural tool to evaluate treatment success.

**Methods:**

A total of 110 patients (20 women, 90 men; mean age 63.7 ± 10.2) with 176 hepatocellular carcinomas were assessed by retrospective analysis of prospectively collected data. The MAM was determined through image fusion of intraprocedural pre- and post-ablation images using commercially available rigid imaging registration software. LTP was assessed in contrast-enhanced CTs or MR scans at 3–6-month intervals.

**Results:**

The MAM was the only significant independent predictor of LTP (*p* = 0.036). For each millimeter increase of the MAM, a 30% reduction of the relative risk for LTP was found (OR = 0.7, 95% CI 0.5–0.98, *p* = 0.036). No LTP was detected in lesions with a MAM > 5 mm. The overall LTP rate was 9 of 110 (8.2%) on a patient level and 10 of 173 (5.7%) on a lesion level. The median MAM was 3.4 (1.7–6.9) mm. The mean overall follow-up period was 26.0 ± 10.3 months.

**Conclusions:**

An immediate assessment of the minimal ablative margin (MAM) can be used as an intraprocedural tool to evaluate the treatment success in patients treated with stereotactic RFA. A MAM > 5 mm has to be achieved to consider an ablation as successful.

**Key Points:**

*• An intraoperatively measured minimal ablative margin (MAM) > 5 mm correlates with complete remission.*

*• MAM is the only significant independent predictor of LTP (OR = 0.7, 95% CI 0.5–0.98, p = 0.036) after stereotactic RFA of hepatocellular carcinoma.*

*• Image fusion using commercially available rigid imaging registration software is possible, even though considerably complex. Therefore, improved (semi-)automatic fusion software is highly desirable.*

## Introduction

In recent years, radiofrequency ablation (RFA) has emerged as a first-line curative treatment approach for patients with small hepatocellular carcinoma (HCC) lesions [1–4]. The 2018 American Association for the Study of Liver Diseases (AASLD) guideline considers RFA and surgical resection as equal treatment options for single tumors < 2.5 cm [5]. Major complications after RFA are rare and treatment-associated mortality is extremely low [6–11].

Technical limitations of the conventional computed tomography (CT)– or US-guided techniques can be overcome by multiple-needle approaches using 3D treatment planning and stereotactic needle placement (termed “stereotactic RFA” or “SRFA”) [12–20].

A crucial point in curative treatments of HCC is the evaluation of the treatment success. In patients treated with surgical resection, the histopathological examination of the surgical specimen gives certainty whether the resection margin is free of cancer cells (R0), cancer cells are present microscopically (R1), or residual gross tumor tissue is present (R2). The resection margin has a significant influence on prognosis of patients with HCC [21–23], and it determines the treatment plan to follow. After radiation therapy, such as SBRT, an immediate assessment of treatment efficacy is not possible. The therapeutic effect can only be seen in subsequent follow-up imaging.

Up until now, it has been similar in RFA. The treatment efficacy of RFA could only be assessed through the absence of residual tumor on 1-month follow-up imaging or through the absence of local tumor progression (LTP) in subsequent follow-up imaging. An intraprocedural, more contemporaneous assessment of treatment success after RFA is highly desirable.

Accumulating evidence has pointed towards the post-ablation safety margin (minimal ablative margin) as a critical determinant of RFA success. In several studies, a minimal ablative margin (MAM) of less than 5 mm has been associated with higher rates of LTP [24–26]. Therefore, we postulate that the assessment of the MAM can be used as an intraprocedural tool to evaluate the treatment success of stereotactic RFA and facilitate further ablation in the same session if needed.

The aim of this study was to assess the minimal ablative margin (MAM) with image fusion of pre- and post-ablation CT images and to evaluate if it can predict LTP independently. Furthermore, we sought to determine a MAM with which a stereotactic RFA can be judged as successful.

## Material and methods

### Patients

This is an institutional review board approved, single-center, retrospective analysis of prospectively collected data from HCC patients referred to stereotactic RFA between January 2009 and February 2016.

In all patients, the treatment plan was established by a multidisciplinary tumor board consisting of hepatologists, oncologists, transplant surgeons, and interventional radiologists. Treatment choice was based on tumor characteristics, Child-Pugh classification, anatomical considerations, and the general patient condition.

Inclusion criteria for stereotactic RFA were as follows: (1) Tumors showed the typical imaging characteristics with hypervascularity in the arterial phase and washout in the delayed portal venous phase on contrast-enhanced CT and were accessible by a percutaneous approach [27]; (2) prothrombin time ratio greater than 50% (prothrombin time with international normalized ratio, G1.7) and platelet count greater than 60,000 cells/mm^3^; (3) absence of portal vein thrombosis and extrahepatic metastases.

Routine pre-operative evaluation of patients with hepatocellular carcinoma included baseline history, physical examination, and serum laboratory tests. Contrast-enhanced CT of the abdomen was performed for HCC diagnosis accordingly to the European Association for the Study of the Liver (EASL) [27]. In addition, chest CT was routinely performed to exclude the presence of pulmonary metastases. All HCCs included in this study were pathologically confirmed by needle biopsy during the stereotactic RFA procedure.

**Fig. 1:**
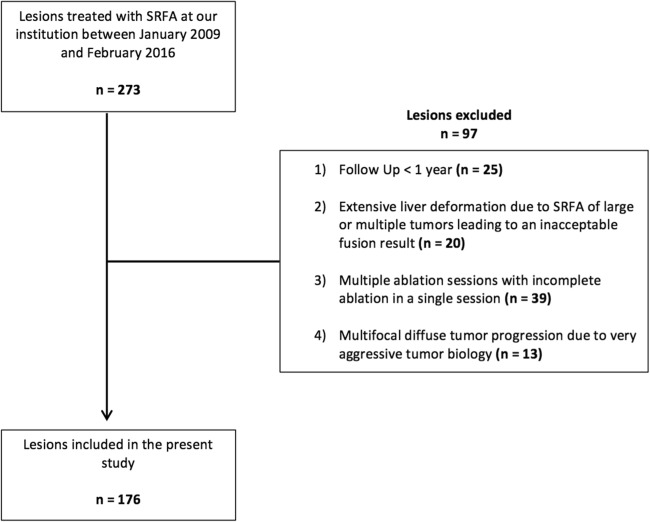
Exclusion criteria for the evaluation of MAM using image fusion leading to 176 HCCs in 110 patients

In total, 146 patients with 273 HCC lesions were identified. Exclusion criteria for retrospective evaluation of MAM were as follows: follow-up < 1 year; extensive liver deformation due to stereotactic RFA of large or multiple tumors leading to an inacceptable fusion result; multiple ablation sessions with incomplete ablation in a single session; multifocal diffuse tumor progression due to very aggressive tumor biology (Fig. 1).

### Stereotactic radiofrequency ablation

The detailed stereotactic RFA procedure has been described elsewhere [12, 13, 19, 20]. Briefly, the procedure is performed in an interventional CT suite under general anesthesia and neuromuscular blockade. The patient is fixed on a CT table by a vacuum bag (Bluebag, Medical Intelligence). Ten to 15 registration markers for image to patient registration (Beekley Spots, Beekley Corporation) are attached to the skin of the thorax and the upper abdomen. Thereafter, a dual-phase contrast-enhanced planning CT (Siemens SOMATOM Sensation Open, sliding gantry with 82 cm diameter, Siemens AG) with 3-mm slice thickness is acquired. Images are obtained 35–40 and 70–80 s after initiation of contrast material injection (100–150 ml of iopromide [Ultravist 370; Schering AG]), representing late arterial and late portal venous phases. All CT images are acquired with the patient in breath hold by temporary tracheal tube disconnection to overcome differences in position due to respiratory motion.

The CT data is then sent to the optical-based navigation system (Stealth Station Treon Plus, Medtronic Inc.). Multiple electrode positions are planned on the multiplanar and reconstructed images in order to cover the entire tumor volume. Following registration, accuracy check, and sterile draping, the ATLAS aiming device (Medical Intelligence) is manually adjusted using the guidance software of the stereotactic navigation system and 15G coaxial needles (Bard Inc.) are introduced through the aiming device to a pre-planned depth. A CT scan for verification of correct needle placement is obtained. A biopsy sample is taken via the coaxial needles using a 16-gauge core biopsy needle. Subsequently, three radiofrequency probes with a 3 cm active tip (Cool-tip, Medtronic) are introduced via the 15G coaxial needles, the latter being retracted to uncover the active probe exposure. At each position, ablations are performed using the switching control mode for up to three probes during the 16-min ablation per cycle. In case of significant increase of impedance (the so-called roll-off effect) the ablation process was terminated. After ablation completion and removal of RFA probes and co-axial needles by tract cauterization, a dual-phase contrast-enhanced CT scan is obtained for complication and ablation assessment.

A complete ablation was defined by a circumscribed non-enhancing area within and/or extending beyond tumor borders. Any areas of abnormally enhanced tissue in the late arterial phase and washout in the delayed phase that were located within or along the margin of the coagulation zone were considered residual tumor.

### Image fusion and evaluation of minimal ablative margin

Computed tomography imaging fusion was performed using commercially available rigid imaging registration software (Syngo.via VB20A, Siemens Healthineers) with automatic registration followed by verification and, if required, manual registration. The arterial phase was utilized as the image of choice for pre-ablation images as the lesions demarcate clearly from the surrounding liver parenchyma which facilitated an accurate evaluation of the MAM. If possible, the late arterial phase was used as the post-ablation image dataset because the landmarks were clearly identifiable and could be easily correlated with the late arterial planning phase.

At first, images were registered automatically by a rigid registration tool included in the software. However, automatic registration was not satisfactory. Through manual correction by multiplanar slight shifting (translation and rotation) in axial, coronal, and sagittal planes referring to well defined intrahepatic structures, a satisfactory fusion was finally achieved. These so-called intrahepatic landmarks were in most cases vessel bifurcation of hepatic arteries or segmental branches of the portal vein. Landmarks close to the lesion, if possible in the same segment, were preferably used to fuse images accurately. In patients with more than one lesion, image registration was repeated for every lesion in other segments if necessary.

**Fig. 2:**
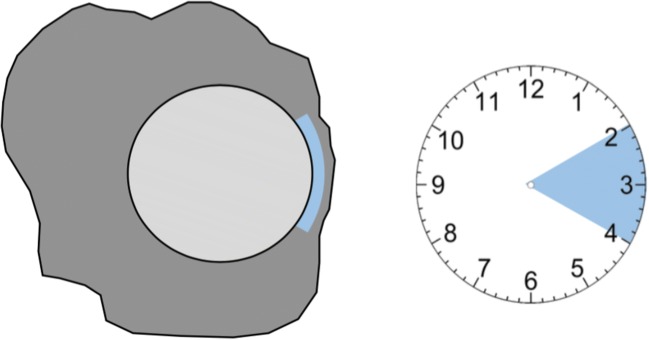
Diagram to illustrate the extension of MAM on a 12-h scale of an analogue watch with lesion (gray), ablation zone (dark gray), and extension of MAM (light blue)

**Fig. 3:**
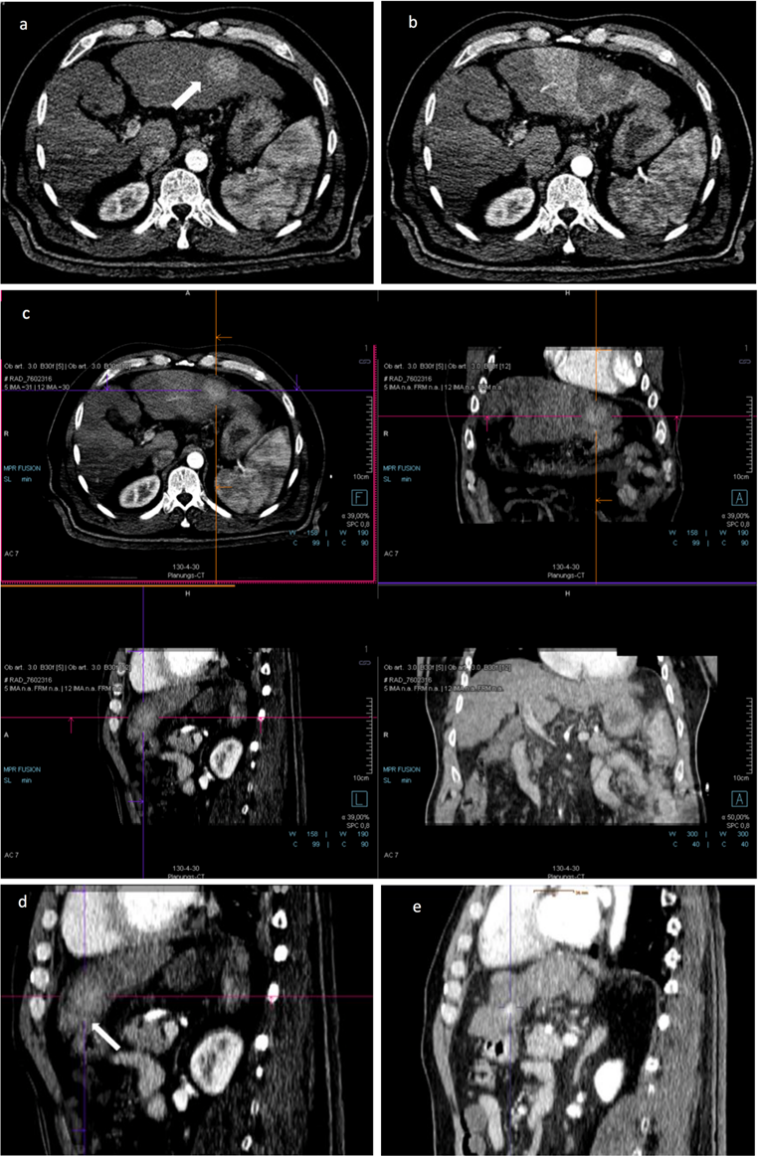
A 63-year-old male patient treated with stereotactic RFA. **a** The arterial phase of pre-SRFA CT scan depicting a single HCC lesion (arrow) with a maximal diameter of 3.4 cm in liver segment II. **b** The arterial phase of post-SRFA CT scan with transient hyperemic rim around the ablation zone. **c** The after fusion of pre- and post-SRFA CT scan with manual registration by slight shifting (translation and rotation) in axial, coronal, and sagittal planes referring to intrahepatic structures such as vessel bifurcations. **d** The sagittal plane of CT image fusion with a MAM of 2.2 mm in clock position 5–6 h (arrow). **e** Local tumor progression after stereotactic RFA: contrast-enhanced CT scan 20.2 months after stereotactic RFA revealing a hypervascular, contrast-enhancing lesion in the arterial phase and with washout in delayed phase that is located immediately adjacent to the ablation zone

**Fig. 4:**
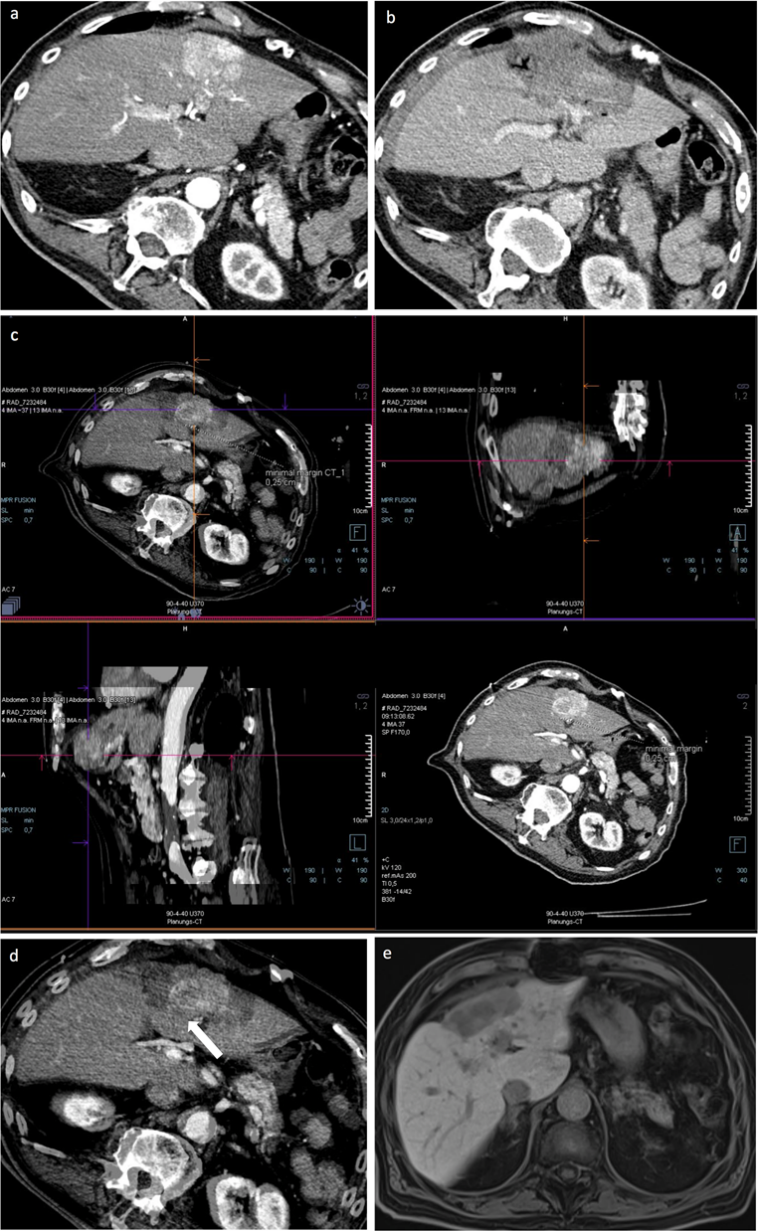
An 88-year-old male patient treated with stereotactic RFA. **a** The arterial phase of pre-SRFA CT scan of a single HCC lesion with a maximal diameter of 6 cm in liver segments II, III, IVa, and IVb. **b** The late portal venous phase of post-SRFA CT scan showing clear demarcation of the ablation zone. **c** The after fusion of pre- and post-SRFA CT scan with manual registration by multiplanar slight shifting (translation and rotation) in axial, coronal, and sagittal planes referring to intrahepatic structures such as vessel bifurcation. **d** The axial plane of CT image fusion with a MAM of 2.5 mm in clock position 6–8 h (arrow). **e** MRI scan 24.9 months after ablation without evidence of LTP and progressively shrinking ablation zone

**Fig. 5:**
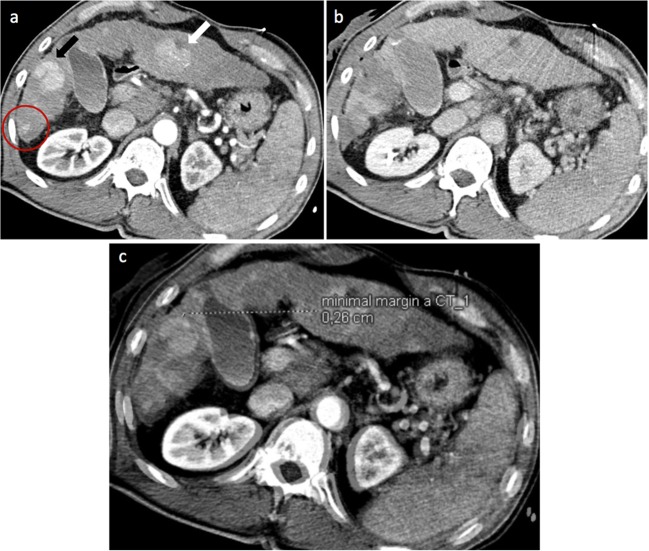
Registration failure of a lesion in a 58-year-old male. **a** The arterial phase of pre-SRFA CT scan showing lesions in liver segment III with 4 cm in diameter (white arrow), in liver segment V with 3.2 cm (black arrow), and in liver segment VI with 1.6 cm (red circle). **b** The late portal venous phase of post-SRFA CT scan with treated lesions in liver segments V and VI. The lesion in liver segment V was treated in a second session 2 months later. **c** The successful fusion of pre- and post-SRFA CT scan with the successful fusion of the lesion in liver segment V (MAM 2.6 mm in clock position 12–2 h). An adequate fusion for the lesion in liver segment VI was not possible due to its subcapsular location and extensive liver deformation

After successful fusion of pre- and post-ablation images, the distances between tumor border and margin of the necrosis zone in the axial, coronal, and sagittal planes were measured. The smallest distance was defined as the minimal ablative margin (MAM). The extension of the minimal ablative margin was described in hours using a 12-h scale of an analogue watch, as illustrated in Fig. 2. An example of successful image fusion and evaluation of MAM is shown in Figs. 3 and 4, while Fig. 5 shows an example of registration failure due to extensive liver deformation.

### Stereotactic RFA imaging response assessment and patient follow-up

After stereotactic RFA, contrast-enhanced follow-up CTs with 3-mm slice thickness (including native, late arterial, late portal, and delayed phases) were performed at 3–6-month intervals. For our study, we followed the treated tumors for a maximum time of 36 months for patients without local tumor progression (LTP). In patients with LTP, follow-up ended with the date of detection of the local tumor progression (i.e., event). In patients with liver transplantation after stereotactic RFA as bridging therapy, the histopathological exam of the explanted liver was used to determine LTP. Residual vital tissue was judged as LTP and follow-up ended with the date of liver transplantation (i.e., event).

Local tumor progression was defined as a newly detected nodular hypervascular lesion with washout in the late portal venous phase immediately adjacent to the ablation zone and detected within 36 months after the intervention (Fig. 3e). Newly detected tumors distant to the ablation zone were defined as distant tumor recurrence.

Both image fusion and evaluation of the minimal ablative margin were conducted blinded regarding the oncological outcome of the patient.

### Statistical analysis

Based on previously published data by Nishikawa et al [28], we performed a sample size calculation in order to determine how many lesions had to be included in the present study to detect a possible difference in local tumor progression between ablations with sufficient (> 5 mm) and insufficient minimal margins (< 5 mm). Assuming a type 1 error of 0.05 and a type 2 error of 0.2 (thus yielding 80% statistical power), we found that a total of 77 lesions had to be analyzed to detect such a difference.

The distribution (parametric/non-parametric) of all variables was assessed using histograms. Baseline characteristics of the patients are expressed as mean ± SD or median with interquartile range, as appropriate. Binary logistic regression (LR) was used to identify independent predictors of local tumor progression. In a second step, Cox regression modeling was utilized to visualize the hazard of LTP over time related to different covariates.

All statistical analyses were performed using SPSS Version 22 (SPSS Inc.). *P* Values < 0.05 were considered statistically significant.

## Results

Baseline characteristics of patients included in our analysis are shown in Table 1.

**Table 1:** Characteristics of 110 patients with a total of 176 HCCs ablated with stereotactic RFA. ^†^Mean ± standard deviation; ^‡^median (interquartile range); *HBV*, hepatitis B virus; *HCV*, hepatitis C virus; *AFP*, α-fetoprotein

Characteristics	Value
Age (years)^†^	63.7 ± 10.2
Gender, *n* (%)	
Female	20 (18.2)
Male	90 (81.8)
Cirrhosis, *n* (%)	95 (86.4)
HBV	6 (5.5)
HCV	19 (17.3)
Fatty liver disease (AFLD and NAFLD)	45 (40.9)
Other	25 (27.7)
Child Pugh, *n* (%)	
Child A	78 (82.1)
Child B	15 (15.8)
Child C	2 (2.1)
AFP (ng/l)^‡^	7 (3.5–24.6)
No. of tumors treated per patient^‡^	2 (1–2)

After exclusion of 97 lesions, 110 patients with 176 HCC treated by stereotactic RFA were finally analyzed in this study (Fig. 4).

The mean overall follow-up period was 26.0 ± 10.3 months. The mean tumor size was 25.2 ± 14.9 mm. Seventy-seven lesions had no specificities regarding their location. The location of the remaining ninety-nine lesions was described as shown in Table 2. The median minimal ablative margin was 3 (2–7) mm. Subdivided into groups, 110 (62.5%) lesions had a MAM of less than 5 mm, whereas sixty-six (37.5%) lesions a MAM greater than 5 mm. Overall LTP rate was 9 of 110 (8.2%) on a patient level and 10 of 173 (5.7%) per ablated HCC. Mean time to LTP was 16.9 ± 9.3 months. The median extension of the minimal margin was 2 h on a 12-h scale of an analogue watch in the corresponding plane (axial, coronal, or sagittal). In 96 (54.5%) cases, a late arterial phase was utilized as a post-ablation image for image fusion, while in the remaining cases, a late portal venous phase had to be used.

**Table 2:** Characteristics of 176 HCCs ablated with stereotactic RFA. ^†^Mean ± standard deviation; ^‡^median (interquartile range); *MAM*, minimal ablative margin; *LTP*, local tumor progression

Characteristics	Value
Tumor size^†^ [range] (mm)	25.2 ± 14.9 [2–83]
Tumor size group, *n* (%)	
< 3 cm	121 (68.8)
3–5 cm	43 (24.4)
> 5 cm	12 (6.8)
Tumor location	
Proximity to gallbladder	4 (2.3)
Proximity to major vessel	32 (18.2)
Proximity to extrahepatic organ	11 (6.3)
Subcapsular	38 (21.6)
Subphrenic	14 (8.0)
No specificities	77 (43.8)
Follow-up in months^†^	26.0 ± 10.3
MAM^‡^ (mm)	3 (2–7)
MAM, *n* (%)	
< 5 mm	110 (62.5)
> 5 mm	66 (37.5)
MAM extension in hours^‡^	2 (1–2)
LTP, *n* (%)	10 (5.7)
Time to LTP in months^†^	16.9 ± 9.3

### Predictors of local tumor progression

Our analysis revealed MAM as the only significant independent predictor of LTP (*p* = 0.036). The *R*^2^ (Nagelkerke) of the model was calculated with 0.17, which translates into a strong effect size (0.45) according to Cohen. For each millimeter increase of the MAM, a 30% reduction of the relative risk for LTP was found (OR = 0.7, 95% CI 0.5–0.98, *p* = 0.036). No other variable such as tumor size or tumor location showed a significant influence on LTP.

The highly significant model of the binary logistic regression with the three possible determinants of LTP is shown in Table 3.

**Table 3:** Binary logistic regression. Model with three possible determinants of LTP. *MAM*, minimal ablative margin; *CI*, confidence interval; *LTP*, local tumor progression

	Odds ratio	95% CI	*p* value	*R*^2^ Nagelkerke
Tumor size	1.023	0.987–1.060	0.222	
MAM	0.700	0.502–0.977	0.036	
Tumor location	0.937	0.663–1.324	0.714	
Model summary			0.012	0.171

**Fig. 6:**
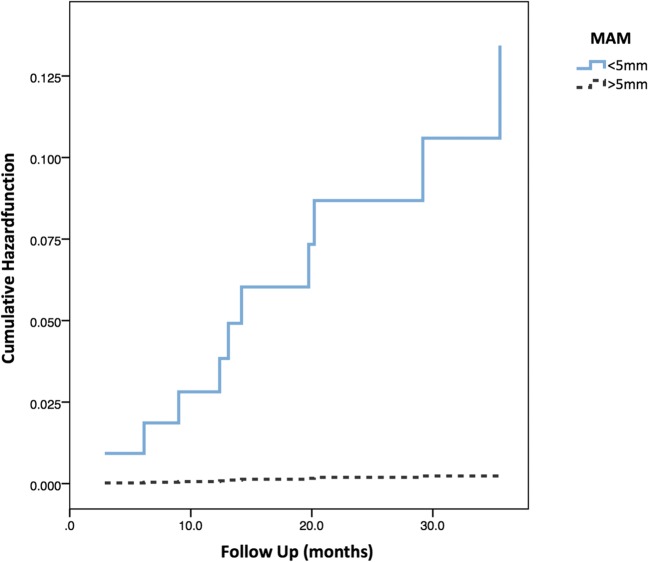
Cox regression with hazard function of LTP over time, subdivided in MAM < 5 mm (blue) and MAM > 5 mm (black)

To visualize the probability of LTP over time, Cox regression was utilized. As the MAM was found to be the only predictor of LTP in binary logistic regression, separate lines were coded for lesions with a MAM > 5 mm and < 5 mm, respectively. No LTP was detected in lesions with a MAM > 5 mm. The results are shown in Fig. 6.

## Discussion

Our data confirm that an ablation with a MAM > 5 mm can be considered successful, due to an extremely low probability of LTP, as already shown in other studies [24–26, 28, 29].

In our analysis, the median MAM was 3 mm and 37.5% (66/176) of the lesions were circumscribed by a MAM > 5 mm. This indicates the enormous efficacy of stereotactic RFA, since in a similar retrospective study with image fusion, a circumscribed MAM > 5 mm after RFA could only be achieved in 2.7% (3/110) of all cases [30].

Our study shows that with image fusion and evaluation of the MAM, the risk of LTP can be significantly reduced, if not avoided altogether, once a circumscribed MAM > 5 mm has been achieved. Under those conditions, we did not observe any case of LTP, which underlines the crucial impact of the MAM on LTP.

In this context, it is important to underline the clinical need to fuse pre- and post-ablation images and determine the MAM immediately after the ablation, such that an ablation can be repeated or continued in locations where a MAM > 5 mm could not be guaranteed.

In previous studies, the MAM was assessed through side-by-side juxtaposition of pre- and post-ablation CT scans with automatic rigid registration software and final manual adjustment [31, 32]; a similar approach is used in our daily clinical practice. This includes automatic mutual information based rigid registration and, in addition, definition of corresponding anatomical landmarks on the different image datasets [12]. However, this method is rather challenging even for very experienced radiologists, considering the need to determine the MAM in three planes in a side-by-side manner. As a result, it contains many potential sources of error.

Our study shows that an image fusion using commercially available rigid imaging registration software is possible and the assessment of the MAM with it can be used as an intraoperative tool to evaluate the treatment efficacy of stereotactic RFA. Nevertheless, a limitation has to be discussed. An appropriate image fusion is considerably complex. Despite getting used to the software and the procedure, a fusion with a lesion demarking clearly from the surrounding liver parenchyma and near intrahepatic landmarks, including the quantitative evaluation of the minimal margin, could not be performed in under 15 min. Therefore, improved (semi-)automatic software is desperately needed, with which an image fusion of pre- and post-ablation CT scans is feasible in only a few minutes and hence applicable in daily clinical practice. An alternative image fusion software platform for volumetric assessment of ablation completeness was recently used in a retrospective study and may be a promising candidate [33]. Still, further investigations are needed before introducing it into daily clinical practice.

Regarding the issue of unsuccessful registration due to extensive liver deformation that encountered in registration of very large or multiple tumors, the implementation of non-rigid registration algorithms may be beneficial. However, further investigation in this field is required.

Unlike previous findings [26, 28, 34], our data revealed the MAM as the only significant independent predictor for LTP. Tumor size, often described as a determinant of LTP in patients treated with RFA, had no significant influence on LTP in our study. This is in line with previous studies about stereotactic RFA, where tumor size did not have any influence on LTP [14, 16, 17, 19]. This can be explained by multiple probe positioning with overlapping ablation zones in stereotactic RFA and the 3D navigated stereotactic planning, which is a crucial distinction to conventional CT- or US-guided ablation. This is a very important finding, given that the AASLD currently considers RFA as an equal treatment to surgical resection only in single tumors < 2.5 cm [5]. Indeed, the mean tumor size in our study was 25.2 ± 14.9 mm and thus, many lesions did not fit the abovementioned criteria. Using stereotactic RFA, it is possible to achieve a curative treatment of multiple lesions and lesions up to 10 cm in diameter [12]. Therefore, from the oncological point of view, stereotactic RFA challenges surgical resection as first line treatment, even in patients with lesions > 2.5 cm.

Tumor location also had no significant influence on LTP. This was surprising, considering important location-related influence factors, like the heat sink effect caused by blood flow at the site of ablation. It is known that this effect can be overcome by increased duration and power of ablation, with ablation probes being preferentially positioned next to the vessel site. In conventional RFA, such accurate probe positioning is not always possible and very challenging due to respiratory motion of the patient; it therefore carries a higher risk for procedure-related complications. As mentioned above, SRFA is performed under general anesthesia with full relaxation, and the probes are positioned in tracheal tube disconnection. This guarantees an exact probe positioning as previously planned and avoids imprecisions due to respiratory motion. Therefore, an ablation is possible even in difficult to reach locations. Clearly, lesions closer than 1 cm to the central biliary structures are a priori not feasible for stereotactic RFA.

Overall, our data confirm the enormous efficacy of stereotactic RFA in selected patients with HCC considering the very low LTP rate of only 5.7%. This finding is in line with a recently published study [15], where the efficacy of stereotactic RFA was evaluated by histopathological examination after bridging therapy for liver transplantation. Complete pathological response in the explanted liver specimen was achieved in 183 of 188 nodules (97.3%), and in 50 of 52 nodules > 3 cm (96.2%). For these reasons, the treatment plan of every patient should be established in a multidisciplinary tumor board with hepatologists, oncologists, transplant surgeons, and interventional radiologists, considering stereotactic RFA a valid curative option [35].

We are well aware of the limitations of our study, as it is a single-center study with retrospective character. Nevertheless, the results are clear and encouraging, justifying specialized training in stereotactic techniques as well as additional costs related to infrastructure.

In summary our study revealed three important findings.

First, an immediate assessment of the minimal ablative margin (MAM) can be used as an intraoperative tool to evaluate the treatment success in patients treated with stereotactic RFA as it appears to be the only significant and independent predictor of LTP.

Second, a MAM > 5 mm has to be achieved to avoid local tumor progression (LTP) and to consider an ablation as successful.

Third, image fusion using commercially available rigid imaging registration software is possible, even though considerably complex. Therefore, improved (semi-)automatic software is desperately needed.

## Abbreviations

AASLD: American Association for the Study of Liver Diseases
CT: Computed tomography
EASL: European Association for the Study of the Liver
HCC: Hepatocellular carcinoma
LTP: Local tumor progression
MAM: Minimal ablative margin
RFA: Radiofrequency ablation
SBRT: Stereotactic body radiotherapy
SRFA: Stereotactic radiofrequency ablation
